# Aggressive Primary Sacral Non-Hodgkin Lymphoma Associated With Systemic Lupus Erythematosus

**DOI:** 10.14740/jmc5328

**Published:** 2026-07-01

**Authors:** Claudia Katiuska Gonzalez-Valdez, Gabriel Galvan-Salazar, Sharon Herrera-Cifuentes, Alejandro Ramos-Alanis, Fernando Pazos-Gomez, Jonas Galindo-Mora, Hector Alva-Sanchez, Rebeca Gil-Garcia

**Affiliations:** a Neurosurgery and Radiosurgery Department, Oncology Centre, Hospital Espanol, Mexico City, Mexico; b Oncology Centre, Hospital Espanol, Mexico City, Mexico; c Pathology Department, Hospital Espanol, Mexico City, Mexico; d Pain Clinic Department, Hospital Espanol, Mexico City, Mexico; e Neurosurgery Department, Hospital Espanol, Mexico City, Mexico; f Institute of Physics, National Autonomous University of Mexico (UNAM), Mexico City, Mexico

**Keywords:** Sacral, Non-Hodgkin lymphoma, Aggressive, Lupus erythematosus

## Abstract

In this case report, we discuss a 36-year-old female patient diagnosed with a rare and aggressive sacral non-Hodgkin lymphoma. The patient presented with a rapidly progressive low back pain radiating to both lower limbs and, following a neurosurgical resection, was diagnosed with sacral non-Hodgkin lymphoma. Sacral involvement is rare among bone pathologies, accounting for only 1–7% of all spinal tumors. The association of this neoplasm with systemic lupus erythematosus at this anatomical site is uncommon and was correlated with an aggressive clinical course in our patient. Magnetic resonance imaging revealed lytic and infiltrative lesions involving the sacrum and L4 on T1- and T2-weighted sequences. Histopathological and immunohistochemical analyses confirmed a diagnosis of diffuse large B-cell lymphoma, expressing CD20, Bcl-6, Bcl-2, CD10, PAX-5, MUM-1, and c-MYC, with a high proliferative index (Ki-67: 90%), and negative for CD3, CK AE1/AE3, CD30, and TdT. During disease progression, the patient developed multiple metastases involving the brain, breast, lung, and submandibular lymph node. Despite treatment with combined chemotherapy and radiotherapy, the aggressive nature of the disease resulted in a poor prognosis.

## Introduction

Among non-Hodgkin lymphomas (NHLs), the most frequent type is diffuse large B-cell lymphoma (DLBCL), accounting for approximately one-third of all NHL cases [1]. The occurrence of primary DLBCL in the sacrum is extremely rare, representing only about 2% of lymphomas, making it an uncommon location [2–4]. In general terms, tumors affecting the sacrum account for between 1% and 7% of all tumors involving the spine [5].

Several studies have linked multiple risk factors with the development of DLBCL. Among the most relevant factors are severe immunodeficiency conditions, such as those in patients with HIV, individuals with immunodeficiency syndromes, organ transplant recipients, and patients with chronic immunosuppression, especially those suffering from rheumatologic diseases. Among the latter, Sjogren’s syndrome, systemic lupus erythematosus, and rheumatoid arthritis stand out. Furthermore, associations have been observed with viral infections such as hepatitis C, Epstein-Barr virus, and Kaposi’s sarcoma, as well as in individuals with metabolic disorders such as obesity and in those exposed to pesticides, herbicides, and other chemical substances [1].

This case report describes the unusual location of a primary lymphoma in a patient with systemic lupus erythematosus, a key risk factor for an aggressive disease.

## Case Report

A 36-year-old woman, with a 2-week history of neuropathic pain in both legs, more intense on the left side, accompanied by paraparesis that prevented walking, urinary incontinence, and localized pain in the sacral region, was admitted in the hospital. Initially, nonsteroidal anti-inflammatory drugs were prescribed, and lumbar and sacral magnetic resonance imaging (MRI) was requested. Given the radiological findings and lack of clinical improvement, hospitalization was decided for further evaluation and treatment.

Neurological examination revealed a conscious and oriented patient with preserved cranial nerves. Muscle strength was evaluated using the Daniels and Worthingham Muscle Grading Scale (0–5). The patient had normal muscle strength in the upper extremities (5/5) and mild weakness in the lower extremities (4/5). Generalized hyperreflexia was detected, along with bilateral but inconsistent Babinski sign, and a positive Lasegue sign in both legs, predominantly on the left. Patrick’s test was positive on the left side, accompanied by pain upon palpation and movement of the left hip. In addition, hypoesthesia was present in the L3, L4, and S1 dermatomes bilaterally, together with severe sacral pain with a score of 8/10 on the visual analogue scale (VAS).

Relevant medical history included systemic lupus erythematosus, two previous acute myocardial infarctions treated with the placement of two coronary stents, an episode of listeria meningitis during the last trimester of pregnancy, neurolupus manifestations, hysterectomy due to uterine fibroids, alveolar hemorrhage, cholelithiasis, and hemolytic anemia.

Simple and contrast-enhanced MRI of the lumbar-sacral spine revealed a diffuse lytic and infiltrative lesion located in the sacrum, left iliac crest, and L4 vertebral body. Mild contrast enhancement was observed in a solid lesion in the left S1 region, along with hyperintensity of the lumbar paravertebral muscles (Fig. 1). Subsequently, an oncological protocol was completed, including upper gastrointestinal endoscopy, colonoscopy, and contrast-enhanced thoracoabdominal computed tomography, with no evidence of metastatic disease. Laboratory tests showed no positive tumor markers, except for elevated C-reactive protein (CRP).

**Figure 1:**
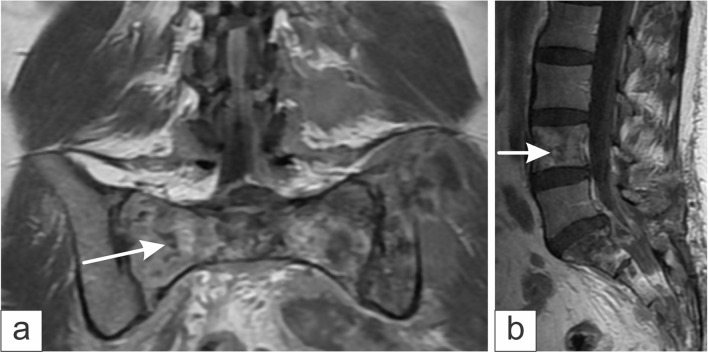
Magnetic resonance imaging obtained using the T2-weighted sequence. (a) Coronal section shows infiltration with multiple lytic and infiltrative lesions at the level of the sacrum (white arrow) and left iliac crest. (b) Sagittal section showing lytic lesions at the level of L4 (white arrow) and the sacrum.

### Clinical course

Neurosurgical intervention was performed, consisting of L5-S1 laminectomy and nerve root decompression. Additionally, decompression and removal of semi-solid epidural tissue located on the left side of S1 were carried out, along with multiple bone and muscle biopsies (Fig. 2). Pathological examination confirmed the presence of NHL, specifically the DLBCL subtype.

**Figure 2:**
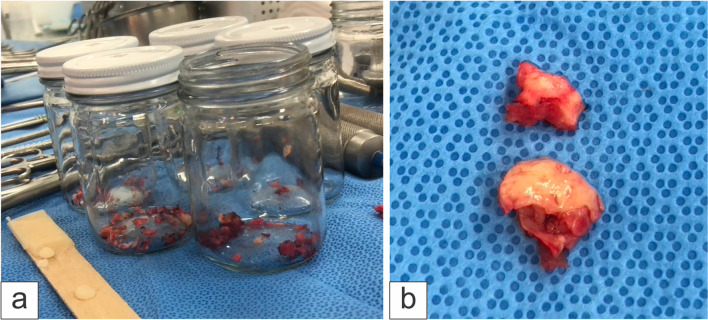
Photographs of (a) sacral bone biopsies and L5 laminectomy, and (b) tissue compressing the extradural S1 region, yellowish in color and soft in consistency.

After the surgical decompression, the patient showed a favorable evolution, with partial pain reduction (VAS 3/10), recovery of urinary continence, and ability to walk. Pain management included a buprenorphine patch and nonsteroidal anti-inflammatory drugs under supervision of the pain clinic.

A laparoscopic cholecystectomy was considered during the same hospitalization due to her recent diagnosis of cholelithiasis; however, this procedure was postponed because 20 days after lumbosacral surgery, the patient experienced recurrence of sacral pain, reaching 8/10 on the VAS. Chemotherapy was initiated, followed by radiotherapy directed to the lumbosacral region and iliac crests. Neurological stability of the lower limbs was maintained, achieving adequate pain control.

Six months after the diagnosis of DLBCL, and 1 month after completing radiotherapy, the patient developed headache, altered level of consciousness, acute confusional state, and frontal lobe symptoms such as disinhibition. She also noted a painful mass in the right breast, prompting her to go to the emergency medical services.

Brain MRI revealed metastases in the frontal, temporal, and occipital regions with perilesional edema (Fig. 3). Chest X-ray showed consolidation in the left lower lobe, while chest CT demonstrated an infiltrate suspicious for pulmonary metastasis (Fig. 4). Mammography revealed bilateral breast fibrosis and a right breast lesion classified as BI-RADS 4.

**Figure 3:**
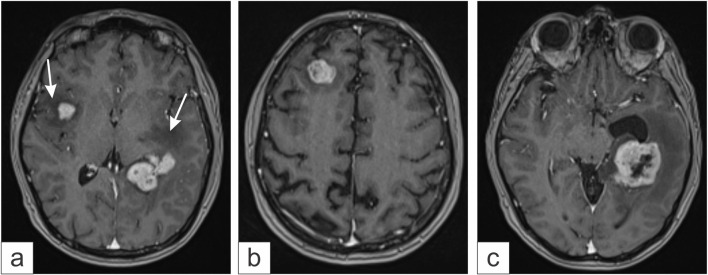
Gadolinium-enhanced brain magnetic resonance imaging. (a) Multiple metastases with perilesional edema (white arrows) are observed. (b) Right frontal metastases. (c) Metastases in the left temporal region.

**Figure 4:**
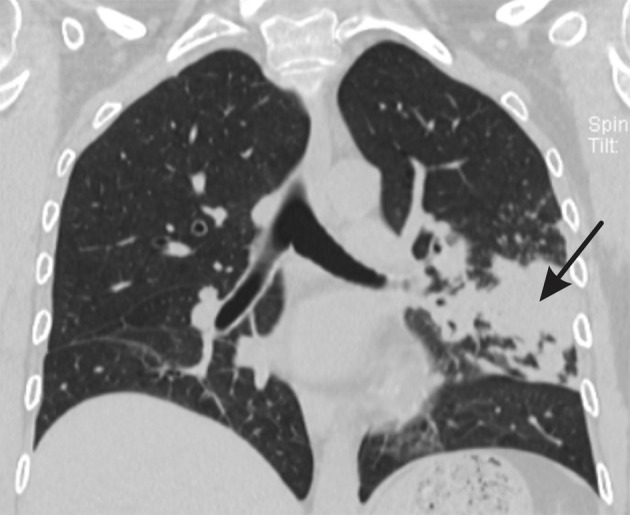
Coronal chest CT scan with lung window showing an infiltrate in the left lower lobe (black arrow), consistent with probable pulmonary metastasis from non-Hodgkin lymphoma.

Whole-brain radiotherapy, bronchoscopy with biopsy and bronchoalveolar lavage, and right breast tumorectomy were performed. Chemotherapy was continued. Histopathological analysis of bronchoscopy samples was inconclusive, whereas the breast tumor showed identical characteristics to the primary sacral lesion.

One month after diagnosis of cerebral and breast metastases, the patient developed seizures, severe gastrointestinal bleeding due to duodenal ulcer and vasculitis, and required intensive care unit admission. After stabilization, she was discharged from the hospital.

During her final hospitalization, 1 month later, the patient presented with severe dyspnea, oxygen saturation of 80%, visual disturbances, elevated lactate levels, persistent sacral pain, and right facial droop, attributed to pulmonary involvement and a new right submaxillary mass. Palliative measures were implemented for pain and gastrointestinal bleeding. Eventually, she developed multiorgan failure, and sedation was initiated.

The total survival after diagnosis was 11 months, characterized by multiple complications and recurrent hospitalizations, confirming the aggressive nature of the disease.

### Surgical treatment, histopathological findings, and microbiological evaluation

An open biopsy and lumbosacral decompression via L5-S1 laminectomy were performed. Samples of bone and muscle were obtained, and resection of solid tissue in left S1 was conducted. The tissue was gray-yellowish and soft, while muscle appeared fragile (Fig. 2).

Pathological analysis confirmed DLBCL (Fig. 5) with markers CD20^+^, Bcl-6^+^, Bcl-2^+^, CD10^+^, Ki67 90%, CD3^−^, PAX-5^+^, CKE1/AE3^−^, MUM-1^+^, CD30^−^, TdT^−^, and cMYC^+^ (Fig. 6). The morphological and immunohistochemistry findings are consistent with a DLBCL, germinal center type by Hans criteria. Molecular testing showed positive cMYC translocation, associated with aggressive behavior and poor prognosis [6, 7]. Muscle and solid tissue samples indicated chronic inflammation. Bronchoscopy samples were inconclusive. Cultures were negative for viruses, bacteria, mycobacteria, and fungi.

**Figure 5:**
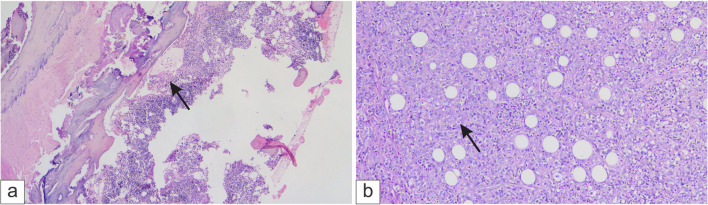
Histological analysis of the trabecular bone. (a) Infiltration by neoplastic lymphoma cells (black arrow) and (b) Diffuse infiltration of large cells (black arrow).

**Figure 6:**
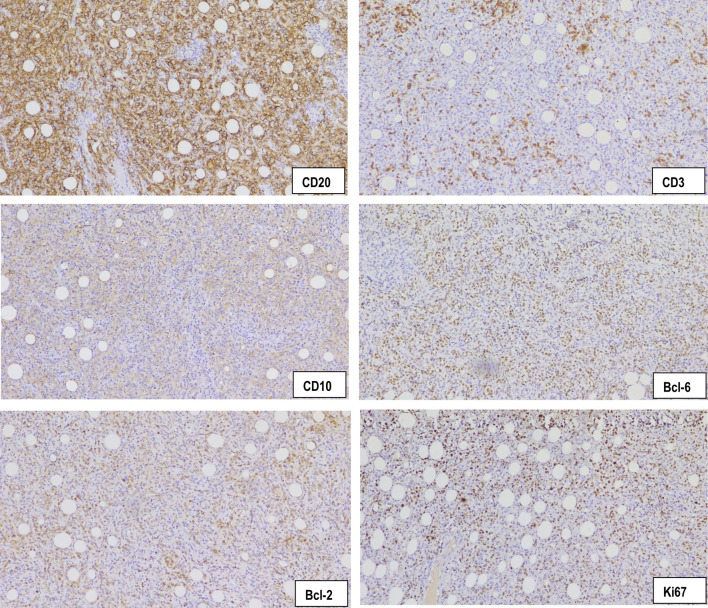
Immunohistochemical analysis of the trabecular bone: CD20, × 40, membranous staining in neoplastic lymphocytes; CD3, × 40, negative in neoplastic lymphocytes expressed in residual T lymphocytes; CD10, 40X, membranous staining in neoplastic lymphocytes; Bcl-6, × 40, nuclear staining in neoplastic lymphocytes; Bcl-2, × 40, membranous staining in neoplastic lymphocytes; Ki67, × 40, nuclear staining in 90% of the neoplastic lymphocytes.

### Oncological treatment

The patient received six cycles of R-CHOP chemotherapy (cyclophosphamide, adriamycin, vincristine, prednisone, and rituximab). In the follow-up MRI persistent lytic and infiltrative lesions in the lumbosacral region and iliac crests were identified. Radiotherapy was administered using intensity modulated radiotherapy (IMRT, 45 Gy in 25 sessions). Whole-brain radiotherapy (36 Gy in 12 sessions) was given for cerebral metastases (Fig. 7).

**Figure 7:**
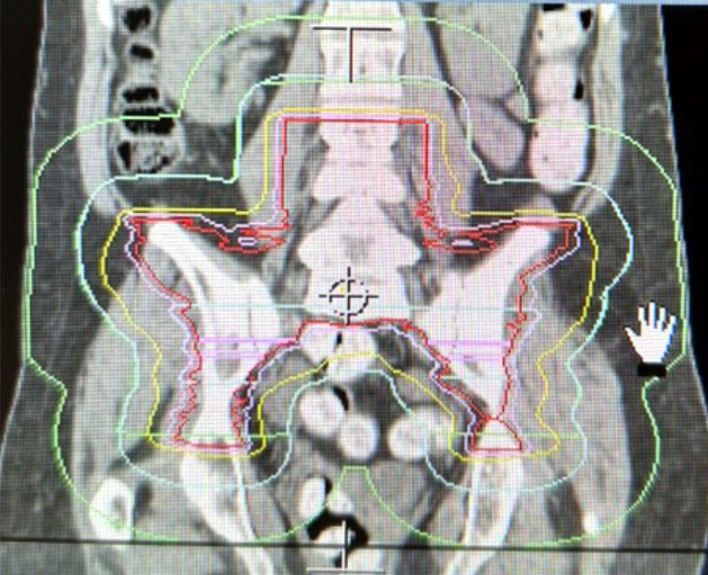
External beam radiotherapy treatment plan using IMRT to the lumbosacral region and iliac crests. A dose of 45 Gy to the target volume, delineated by the red isodose curves was delivered.

## Discussion

We present a case of a woman who initially developed lumbosacral radiculopathy, low back pain, urinary incontinence, and severe pain in the sacral region. She was ultimately diagnosed with non-Hodgkin B-cell lymphoma, diffuse large B-cell subtype, with immunophenotypic markers CD20^+^, Bcl-6^+^, Bcl-2^+^, CD10^+^, Ki67 at 90%, CD3 negative, PAX-5 positive, CKE1/AE3 negative, MUM-1 positive, CD30 and TdT negative, and cMYC positive, with initial presentation in the sacral region.

In this case, direct open bone biopsy was chosen to expedite diagnosis and provide timely treatment, considering the rapid progression of symptoms and the short period since onset. Likewise, decompression of the left L5 lumbar and S1 sacral nerve roots was essential to prevent further neurological deterioration, promote functional recovery, and achieve pain control.

According to Uday and Gupta, fine-needle aspiration biopsy offers a specificity of 84% and 93%, respectively [8, 9]. However, to avoid the need for repeating this type of procedure, as reported in several studies [10, 11], open bone biopsy was performed, allowing for faster diagnostic confirmation. The availability of a specialized histopathology team experienced in the evaluation of bone tissue is essential, as proper processing, staining, and accurate interpretation of samples require meticulous technique and careful handling.

The patient had a diagnosis of systemic lupus erythematosus with an 18-year disease course and had received various immunosuppressive treatments, including methotrexate, azathioprine, sulfasalazine, and prednisone at different stages of her illness. This history represented a significant risk factor for the development of diffuse large B-cell non-Hodgkin lymphoma, as her immune system remained chronically compromised. The coexistence of lupus also favored the development of pulmonary and cardiac complications, as well as unusual infections, including Listeria monocytogenes and meningitis. All these factors reinforce the association between lymphoma and rheumatologic diseases. The unusual aspect of this case lies its appearance in the sacral region, a location with an incidence of only 2–3% in the sacrum and spine [12]. Typically, this type of hematologic malignancy presents in lymph node chains, liver, spleen, gastrointestinal tract, skin, or long bones.

The Ki67 index of 90% reported in this patient corresponds to a very high neoplastic cell proliferation rate. A high index usually indicates potential sensitivity to treatment. However, the presence of cMYC positivity, Bcl6 positivity, and MUM-1 negativity confers a limited therapeutic response and a poor overall prognosis [6, 13, 14].

### Conclusions

This case report describes a woman in her 40s diagnosed with aggressive primary sacral DLBCL associated with long-standing systemic lupus erythematosus. Open bone biopsy and surgical decompression were essential for rapid diagnosis and neurological recovery. Her 18-year history of lupus and prolonged immunosuppressive therapy represented a significant risk factor. The unusual sacral location, high Ki67 index, and positive cMYC translocation explained the aggressive clinical course and unfavorable prognosis.

Due to the rarity of this condition, no standard treatment exists. In patients with multiple risk factors and long-standing autoimmune disease, survival is significantly reduced. Molecular studies are essential to guide prognosis and treatment strategies, including advanced therapies such as immunotherapy and autologous stem cell transplantation.

### Learning points

This case report highlights the need to confirm the diagnosis of the non-Hodgkin sacral lymphoma. This is achieved more quickly and precisely with surgery as opposed to a fine-needle aspiration biopsy. In addition, it is important to consider lymphoma in patients with rheumatologic diseases and atypical neurological symptoms, as the immunosuppression associated with systemic lupus erythematosus may predispose them to more aggressive presentations of hematologic neoplasms and a poor patient response to treatment.

## Data Availability

The authors declare that data supporting the findings of this study are available within the article upon reasonable request.

## Abbreviations

CRP: C-reactive protein
CT: computed tomography
DLBCL: diffuse large B-cell lymphoma
HIV: human immunodeficiency virus
IMRT: intensity modulated radiotherapy
MRI: magnetic resonance imaging
NHL: non-Hodgkin lymphoma
R-CHOP: cyclophosphamide, adriamycin, vincristine, prednisone, and rituximab
VAS: visual analogue scale

## References

[1] Wang SS (2023). Epidemiology and etiology of diffuse large B-cell lymphoma. Semin Hematol.

[2] Chigurupati SV, Shukla M, Pandey M (2021). Primary sacral non-Hodgkin's lymphoma: report of a case and systematic review of literature. World J Surg Oncol.

[3] Amonkar AP, Mallaiah B, Musthafa FB (2017). Primary lymphoma of the sacrum- A rare entity. Clin Oncol.

[4] Epelbaum R, Haim N, Ben-Shahar M, Ben-Arie Y, Feinsod M, Cohen Y (1986). Non-Hodgkin’s lymphoma presenting with spinal epidural involvement. Cancer.

[5] Garcia-Ortega DY, Clara-Altamirano MA, Gomez-Pedraza A, Martinez-Said H, Maciel-Miranda A, Caro-Sanchez CHS, Cuellar-Hubbe M (2018). Tumores primarios de sacro: analisis de resultados y complicaciones. Acta Ortopedica Mexicana.

[6] Shimada A, Sugimoto KJ, Wakabayashi M, Imai H, Sekiguchi Y, Nakamura N, Sawada T (2013). Primary sacral non-germinal center type diffuse large B-cell lymphoma with MYC translocation: a case report and a review of the literature. Int J Clin Exp Pathol.

[7] Savage KJ, Johnson NA, Ben-Neriah S, Connors JM, Sehn LH, Farinha P, Horsman DE (2009). MYC gene rearrangements are associated with a poor prognosis in diffuse large B-cell lymphoma patients treated with R-CHOP chemotherapy. Blood.

[8] Shergill U, Vitkovski T, Stoffels G, Klein M, Gimenez C, Laser A, Cocker R (2017). Fine-needle aspiration biopsy of lytic bone lesions: an institution's experience. Diagn Cytopathol.

[9] Gupta S, Takhtani D, Gulati M, Khandelwal N, Gupta D, Rajwanshi A, Gupta S (1999). Sonographically guided fine-needle aspiration biopsy of lytic lesions of the spine: technique and indications. J Clin Ultrasound.

[10] Perez Abela AL, Alvarez Osuna RM, Acosta Collado F, Canadas del Castillo A, Martinez Montes JL, Godoy Abad N (2001). Primary osseous lymphoma of the spine. Rev S and Traum y Ort.

[11] Liu JK, Kan P, Schmidt MH (2003). Diffuse large B-cell lymphoma presenting as a sacral tumor Report of two cases. Neurosurg Focus.

[12] Graus F, Arino H, Dalmau J (2014). Paraneoplastic neurological syndromes in Hodgkin and non-Hodgkin lymphomas. Blood.

[13] Johnson NA, Savage KJ, Ludkovski O, Ben-Neriah S, Woods R, Steidl C, Dyer MJ (2009). Lymphomas with concurrent BCL2 and MYC translocations: the critical factors associated with survival. Blood.

[14] Visco C, Tzankov A, Xu-Monette ZY, Miranda RN, Tai YC, Li Y, Liu WM (2013). Patients with diffuse large B-cell lymphoma of germinal center origin with BCL2 translocations have poor outcome, irrespective of MYC status: a report from an International DLBCL rituximab-CHOP Consortium Program Study. Haematologica.

